# Neurotrophic and immunomodulatory effects of olfactory ensheathing cells as a strategy for neuroprotection and regeneration

**DOI:** 10.3389/fimmu.2022.1098212

**Published:** 2022-12-19

**Authors:** Simona Denaro, Simona D’Aprile, Cristiana Alberghina, Anna Maria Pavone, Filippo Torrisi, Sebastiano Giallongo, Lucia Longhitano, Giuliana Mannino, Debora Lo Furno, Agata Zappalà, Rosario Giuffrida, Daniele Tibullo, Giovanni Li Volti, Nunzio Vicario, Rosalba Parenti

**Affiliations:** ^1^ Section of Physiology, Department of Biomedical and Biotechnological Sciences, University of Catania, Catania, Italy; ^2^ Section of Biochemistry, Department of Biomedical and Biotechnological Sciences, University of Catania, Catania, Italy; ^3^ Department of Chemical, Biological, Pharmaceutical and Environmental Sciences, University of Messina, Messina, Italy

**Keywords:** OECs, immunomodulation, neurotrophic factors, intercellular communication, neuroregeneration

## Abstract

Accumulating evidence sustains glial cells as critical players during central nervous system (CNS) development, homeostasis and disease. Olfactory ensheathing cells (OECs), a type of specialized glia cells sharing properties with both Schwann cells and astrocytes, are of critical importance in physiological condition during olfactory system development, supporting its regenerative potential throughout the adult life. These characteristics prompted research in the field of cell-based therapy to test OEC grafts in damaged CNS. Neuroprotective mechanisms exerted by OEC grafts are not limited to axonal regeneration and cell differentiation. Indeed, OEC immunomodulatory properties and their phagocytic potential encourage OEC-based approaches for tissue regeneration in case of CNS injury. Herein we reviewed recent advances on the immune role of OECs, their ability to modulate CNS microenvironment *via* bystander effects and the potential of OECs as a cell-based strategy for tissue regeneration.

## 1 Introduction

**Figure 1:**
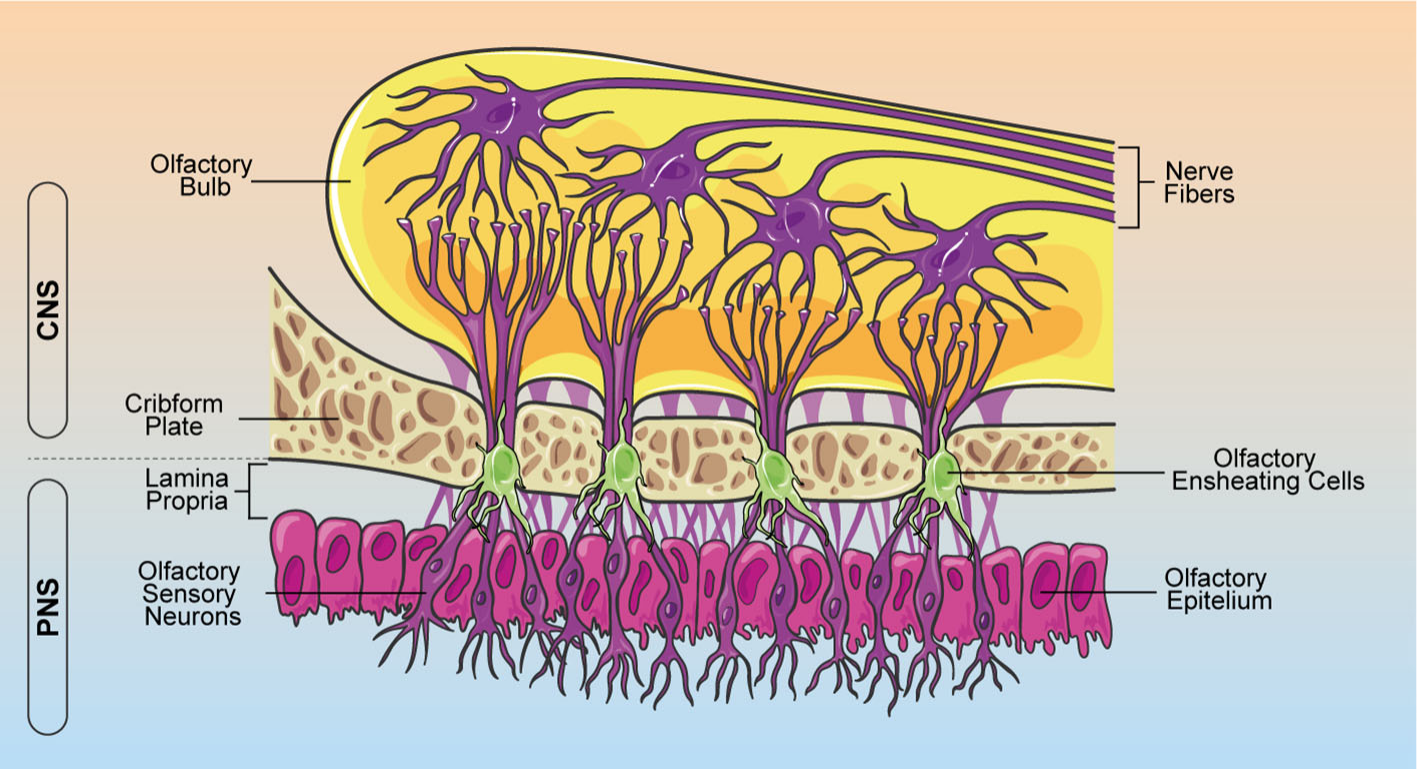
Schematic representation of OEC localization within the olfactory system. OECs ensheath bundles of olfactory receptor axons along their course through the lamina propria in the PNS. Olfactory nerves and their associated OECs cross through the cribform plate into the CNS, making connections with the olfactory bulb. OEC, olfactory ensheathing cells; CNS, central nervous system; PNS, peripheral nervous system.

During the last decades, increasing evidence support the hypothesis that glial cells are important players in crucial aspects of neurogenesis, neuronal functions and diseases (1, 2). Indeed, glial cells guide neuronal migration during development, participate in synaptic formation and plasticity, regulate vasculature and blood–brain barrier (BBB), modulate neuroimmunity, and support neural regeneration (1–3). The term *glia*, from the Greek “γλíα”, meaning “glue”, was originally assigned assuming that these cells were responsible to keep neural cells together. In the adult central nervous system (CNS) three main types of glial cells can be distinguished: astrocytes and oligodendrocytes, deriving from neural crest, and microglia, which originate from the myeloid lineage. In the peripheral nervous system (PNS), Schwann cells represent the main class of glia. Olfactory ensheathing cells (OECs) are a type of specialized glia cells, restricted to the olfactory system, which play a crucial role in olfactory development and regeneration (4–6). Indeed, the olfactory system has a unique neurogenic niche where unlike most regions of the nervous system, olfactory sensory neurons retain a lifetime regeneration potential (4, 5). Since the olfactory neuroepithelium is in direct contact with the external environment, it has evolved a remarkable ability to recruit sensory neurons during normal cell turnover or after traumatic olfactory nerve injury (7, 8). This unique feature is now widely attributed to the presence of OECs, able to wrap olfactory axons and support olfactory receptor neurons turnover and axonal regeneration (9–11). OECs perform their axon growth-promoting properties and provide structural support by extending thin processes that envelop group of axons as an insulator (**Figure 1**) (12, 13). Moreover, when new olfactory sensory neurons are generated from stem cells in the olfactory epithelium, OECs establish functional connections along the olfactory neuroaxis (8, 14).

In contrast to neural crest-derived PNS glia and neural tube-derived CNS glia, OECs have generally been thought to originate from the olfactory placode (15). However, several studies show that the olfactory placode arises from ontogenetically heterogeneous sources of cells and OECs derive from neural crest, like Schwann cells (16–18). These cells are located in the lamina propria of the olfactory mucosa, as well as the outer layers of the olfactory bulbs, the inner and outer nerve fiber layers (**Figure 1**) (19).

OECs share many properties with Schwann cells and astrocytes. They express some typical markers such as the p75 neurotrophic receptor (p75NTR), the polysialylated form of neural cell adhesion molecule (PSA-NCAM), and, like astrocytes, they express the glial fibrillary acid protein (GFAP), and the S100 proteins (20, 21). Furthermore, OECs are able to secret high level of growth factors, such as nerve growth factor (NGF), basic fibroblast growth factors (bFBF), brain derived neurotrophic factor (BDNF), glial derived neurotrophic factor (GDNF), ciliary neurotrophic factor (CNTF), neurotrophins NT4, NT5 and neuregulins, which exhibit important functions as neuronal supporting elements (13, 22–24).

In recent years, significant advances have been made in cellular-based therapies, which focus on the restoration, regrowth or replacement of damaged or dysfunctional cells, tissues and organs, in order to treat neurodegenerative diseases (25) and CNS injuries (26–28). Moreover, cell-based approaches, including OEC grafts, have been reported to induce beneficial effects in spinal cord injury (SCI) models. In addition to neuroprotective mechanisms, axon regeneration and remyelination were observed, leading to significant sensory and locomotor functions amelioration (29–31). Thus, OEC transplantation is proposed as a potential therapeutic strategy for SCI, due to their unique characteristics, such as anti-neuroinflammation, growth-promoting factor secretion, and debris clearance activity. However, there is a lack of in-depth studies focusing on the phagocytic function of these cells, particularly the molecular and cellular mechanisms involved in this intricate process and on the synergistic effects with neural and mesenchymal stem cells (MSCs) in improving cell differentiation. Exploring these unique features will lead to a better understanding of the role of OECs in development and regeneration and will identify how the use of OECs can be optimized for neural regeneration therapies. These approaches may benefit from accumulating evidence pointing out a significant role of checkpoint therapy in inducing regeneration upon CNS injury (32). Herein we reviewed the current knowledge about the immunomodulatory and anti-inflammatory properties of OECs in neuroinflammation, neurodegeneration and during stem cell differentiation. Owing to the strong pro-regenerative properties of OECs, and their unique ability to promote stem cell differentiation, we explored the potential of OEC transplantation for tissue regeneration.

## 2 Immune role of OECs

The olfactory system is continuously exposed to various pathogens since the primary olfactory neurons are in direct contact with the external environment (7, 8). However, most cases of CNS infections do not occur through the olfactory system. In this scenario OECs play a crucial role in protecting CNS structures. Specifically, they participate in innate immune responses, secrete immunoregulatory molecules and exert their phagocytic activity thus maintaining microenvironmental homeostasis, supporting neuron survival and axonal growth (33–35).

### 2.1 Phagocytic activity of OECs

CNS lesions are characterized by neuronal degeneration and death, and by the persistence of cellular and myelinated debris that create an adverse environment for neural survival, germination of neurites and renewal of neurons (36, 37). Since olfactory receptor neurons renew themselves throughout lifetime, a large amount of apoptotic debris is generated continuously (35). Several studies support phagocytic functions of OECs throughout life (35, 38) especially following injury (38, 39). In fact, by switching from a resting state to a phagocytic phenotype to remove axonal debris and bacteria, they protect the olfactory nerve from microbial infections (35, 40, 41). A combination of morphological and phenotypic changes distinguishes reactive OECs from their resting state, including cytoskeletal hypertrophy and rearrangement (34, 42). However, the identification of specific molecular markers capable of discriminating between quiescent and reactive OECs could better elucidate the molecular mechanisms underlying their activation.

While Schwann cells participate in debris removal mainly by increasing the secretion of several pro-inflammatory molecules, thus recruiting professional phagocytes, including macrophages and neutrophils (43), OECs operate differently (38, 44). Wright et al. showed that OECs repel macrophages in co-culture, by expressing the macrophage migration inhibitory factor (MIF), which would explain the absence of macrophages in the olfactory nerve bundles (45).

*In vitro* studies reported that OECs possess several phagocytic-related receptors, including toll-like receptor 4 (TLR4), phosphatidylserine and mannose receptors (34, 46, 47). Particularly, during apoptosis, olfactory neurons display the “eat me” signal phosphatidylserine, recognized by OEC phosphatidylserine receptor, leading to the engulfment of apoptotic and necrotic cell debris (33, 44). Milk fat globule-EGF factor 8 (MFGE-8), which interacts with integrin receptors (48), is a bridging molecule that participates in several cell surface-mediated regulatory events. Li et al. demonstrated *in vitro* that OECs express MFGE-8 when apoptotic debris is added to the culture (49). Moreover, OECs have been reported to adopt a “microglia-like” phenotype showing high levels of CD11 expression after their transplantation into the X-irradiated spinal cord of female Sprague Dawley rats (50). However, *in vitro* immunolabelling of OECs has revealed that they do not express this microglial marker in physiological conditions (34). Interestingly, Nazareth et al. reported that OECs produce less pro-inflammatory cytokines, compared to Schwann cells and macrophages when exposed to necrotic bodies (37). Conversely, some anti-inflammatory cytokines, such as interleukin-10 (IL-10) and transforming growth factor beta (TGF-β) promote OEC phagocytic activity (49).

In summary, the phagocytic activity of OECs plays a crucial role in creating a favorable environment to promote neuronal turnover, aiding the overall process of neuronal regeneration. Hence, this peculiar feature of OECs may be particularly useful for neural repair therapies including their transplantation after SCI.

### 2.2 OEC-mediated effects during neuroinflammation

As abovementioned, OECs show several unique properties of inflammatory cells, allowing them to modulate immune responses and neuronal pro-regenerative processes. Overall, inflammation is thought to hinder cell differentiation and regeneration but, although OECs are able to secrete a range of pro-inflammatory cytokines and chemokines after injury or infections, they simultaneously promote nervous regeneration.

**Figure 2:**
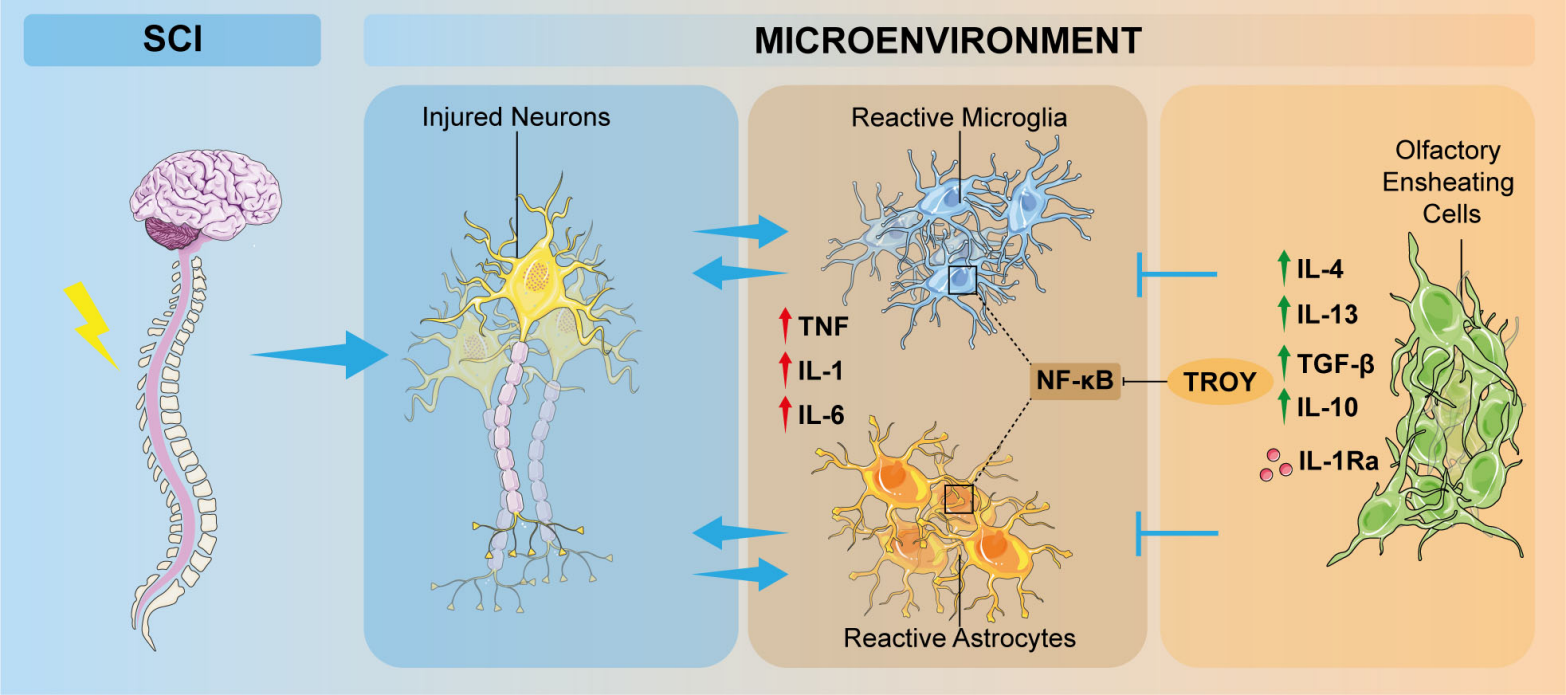
Schematic overview of the involvement of OECs in inflammation modulation after SCI. Neuronal damage induces pathological increasing of inflammatory responses, which promotes microglia polarization from a resting state to a M1-phenotype and astrocyte activation. OECs are able to modulate these inflammatory events by interacting directly or indirectly with microglia and astrocytes, thus ameliorating the detrimental condition of the altered microenvironment. OEC, olfactory ensheathing cell; SCI, spinal cord injury; TNF, tumor necrosis factor; IL, interleukin; TGF-β, transforming growth factor β, IL-1Ra: interleukin-1 receptor antagonist.

Following SCI, resident immune cells, including microglia and astrocytes, are activated by injured-released inflammatory stimuli (51). Indeed, the microenvironment of lesioned CNS switch towards pro-apoptotic and anti-regenerative milieu. Particularly, inflammatory responses in SCI are mainly mediated by pro-inflammatory cytokines and chemokines secreted by reactive astrocytes and microglia. In this scenario, M1-polarized microglia induces astrocyte activation, resulting in chondroitin sulfate proteoglycan (CSPG) deposits and astrocytic scar formation, which limits the spread of inflammation but at the same time hampers axon regeneration (52). Concomitantly, glial cell activation causes the release of specific chemokines and pro-inflammatory cytokines, including IL-1, IL-6, and TNF. These cytokines, by activating their respective cascades, amplify inflammatory responses, alter the microenvironment and promote cell death, therefore blocking axonal regeneration (**Figure 2**) (53, 54). As a result, inflammatory response induces secondary tissue damage with detrimental consequences to neural tissue and its functions (55). In general terms, inflammatory response maintains a dynamic balance of pro-inflammatory and anti-inflammatory cytokine release; therefore, understanding the modulation of the inflammatory response mediated by OECs, could be a successful strategy to improve neuronal functional outcome after CNS injury.

OECs are reported to express chemokines/cytokines and their cognate receptors, such as chemokine (CXC motif) ligand 1 (CXCL1), a neurotrophic chemoattractant, which may have a role during embryogenesis or after OECs transplantation in the injured site, CXCL12, CXCL4, chemokine (CX3C motif) ligand 1 (CX3CL1) (56) that have been proven to play pivotal roles in neuroinflammation, acting as a signaling factor for the recruitment of neutrophilis and various leucocytes (57). The inflammatory monocyte chemotactic protein 1 (MCP-1), and its receptor CCR2 specifically mediates monocytes chemotaxis, which results in the recruitment of macrophages to the site of injury. Moreover, nuclear factor kappa B (NF-κB)-mediated signaling pathway, responsible for microglia and astrocytes activation after SCI, is activated by TROY, a member of the tumor necrosis factor (TNF) receptor superfamily, which has been detected *via in situ* hybridization and immunohistochemistry investigations in the olfactory system (58). OECs or OEC-released molecules are able to inhibit NF-κB activation, so exerting a neuroprotective role after CNS injury. OECs also release several signaling molecules, such as TNF and IL-1β, to recruit macrophages, thus modulating inflammation and neurodegeneration (14, 44, 59). In this context, OECs could modulate microglia-astrocyte responses by secreting anti-inflammatory cytokines such as IL-4, IL-10, IL-13 and TGF-β, capable to downregulate the pro-inflammatory factors IL-1β, TNF and IL-6 (**Figure 2**) (60–62). A recent study showed that IL-1α and IL-1β, which are significantly involved in inflammatory responses, were down-regulated after OEC transplantation at the injury site. This response is probably related to IL-1 receptor antagonist (IL-1Ra) mechanism, which is a competitive inhibitor of IL-1 by binding to its receptor (**Figure 2**). Therefore, OECs, reaching the site of the lesion, are subjected to pro-inflammatory factors released by the activated microglia, and secrete IL-1Ra in response, thus reducing microglial activation and pro-inflammatory factor production and limiting microglia-mediated pro-inflammatory cytokine release (63).

It is worth noticing that the abovementioned OEC-derived anti-inflammatory factors participate in modulating cell survival, proliferation and migration, thus reducing glial scar and promoting regeneration after SCI (64). IL-4 and TGF-β have a direct impact on neural survival given their modulatory effects on acute and chronic immune cell responses and on their expression of detrimental molecules including nitric oxide (NO), reactive oxygen species (ROS), caspase and their secretion of neurotrophins (65).

Taken together these findings suggest that OECs delay the activation of microglia or macrophages and reduce the peak of the immune response, leading to neuroprotection against inflammatory damage.

## 3 OEC bystander effects on cell fate and differentiation

The ability of OECs in regulating neuroprotection is enhanced by the release of several protective factors in the microenvironment as OEC-conditioned medium (OEC-CM) promotes the differentiation of neural stem cells (NSCs) (66). Specifically, using OEC-CM, it has been shown that soluble factors larger than 30 kDa, which are secreted by OECs, promote migration, differentiation and maturation of NSCs within 7 days. By immunocytochemical analysis, it has been shown that NSCs in contact with OEC-CM, exhibited an up-regulation of neurofilament (NF), beta-III-tubulin (TUJ1), GFAP and a down-regulation of nestin, suggesting a differentiation of NSCs toward neuronal and astrocytic lineages. In addition, the presence in NSCs of synapsin-1, which is involved in the neurotransmitter release mechanism, has also been demonstrated, supporting the effect of OEC-CM in driving and/or stimulating neuronal differentiation. This study also claims that differentiation of NSCs, promoted by OECs, also occurs through indirect contact (67). OECs also exert their trophic effects directly through the secretion of factors involved in neurogenesis, neural differentiation and response, including both NGF and BDNF, small proteins including neurturin (NTN), CNTF, GDNF (68, 69), and heavier soluble factors including secreted protein acidic and cysteine rich (SPARC), sonic hedgehog protein (SHH), matrix metalloproteinase 2 (MMP 2), fibronectin, and laminin (70–72) (**Table 1**). Moreover, TGF-β3 secreted by OECs is involved in the regulation of neuronal differentiation, negatively regulating Yes-associated protein (YAP) (76). The potential of OECs to induce differentiation of NSCs into neurons has also been demonstrated by functional electrophysiological studies that showed that NSC-derived neurons exposed to OEC-CM acquire active electrophysiological properties, expressing sodium and potassium channels suitable for onset of action potentials similar to primary neuronal cells (66). *In vivo* studies demonstrated that OECs are able to promote NSC differentiation into dopaminergic neurons or cholinergic neurons, pointing out that OECs can induce NSC differentiation toward a specific neuron subtype (77, 78). OEC-induced effects would be exerted by influencing Wnt/beta-catenin signaling pathway, which is important in the proliferation and self-renewal of adult NSCs (79, 80). Indeed, it was shown that CM from Wnt-activated OECs (wOEC-CM) stimulates the proliferation and differentiation of NSCs, by increasing the percentage of Ki67/Sox2 double positive cells, maintaining Nestin expression under differentiation condition, but also stimulating NSC differentiation into Tuj1-positive neurons (81).

**Table 1:** OEC released factors involved in neural differentiation and neurogenesis.

Factors	Molecular weight	Functions	References
**Brain Derived Neurotrophic Factor (BDNF)**	26.7 kDa	Involved in the promotion of Schwann cell migration	(69)
**Ciliary Neurotrophic Factor (CNTF)**	22.9 kDa	Support neurogenesis	(67)
**Fibronectin**	440 kDa	Promote neural progenitor cell migration	(73)
**Laminin**	400 kDa	Involved in neural progenitor cell differentiation	(74)
**Matrix Metalloproteinase 2 (MMP2)**	67 kDa	Important for neural cell migration	(75)
**Nerve Growth Factor (NGF)**	26.7 kDa	Involved in the promotion of Schwann cell migration	(69)
**Neurturin (NTN)**	23.6 kDa	Support neurogenesis	(67)
**Sonic Hedgehog protein (Shh)**	67 kDa	Induce NSC differentiation into neurons	(72)
**Secreted Protein Acidic and Cysteine Rich (SPARC)**	43 kDa	Implicated in neural differentiation and in neurite extension	(70)

Many reports have shown that hypoxic preconditioned stem cells survive longer, exhibiting an efficient neuronal differentiation and showing enhanced paracrine effects (82, 83). Wang et al. demonstrated that CM from hyperthermia-conditioned OECs induces NSC neural differentiation more efficiently, thanks to the upregulation of HIF-1α, leading to synergistic effects that improve differentiation (84). By using OEC-CM under hypoxic condition, olfactory mucosa MSCs (OM-MSCs) are stimulated to differentiate into dopaminergic neurons. Specifically, OEC-CM under hypoxia upregulates transcriptional factors mediated by HIF-1α and it is involved in the development of dopaminergic neurons from OM-MSCs (85).

MSCs, including adipose tissue-derived MSCs (ASCs), are a type of non-hematopoietic stem cells which under appropriate conditions can give rise to several precursors (86–90). OEC-CM is also implicated in the differentiation ASCs toward a neuronal phenotype (91). ASCs treated with OEC-CM expressed markers of progenitor and mature neurons, including Nestin, protein gene product 9.5 (PGP 9.5), and microtubule-associated protein 2 (MAP2) in a time-dependent manner and exhibited neuron like morphology, while they were negative for GFAP and A2B5, markers of astrocytes and oligodendrocytes, respectively (92). In addition, although a significant increase of Nestin, PGP 9.5, Synapsin I, and GFAP was reported, MAP2 was identified as the most representative, thus suggesting a greater tendency toward the neuronal phenotype (93). This result is confirmed by another study where a neural-like connexin expression was induced in ASCs after OEC-CM treatment (94–98). On the other hand, when ASCs were co-cultured with OECs using 3D collagen scaffolds, they differentiated into cells with OEC-like morphology and were reported to be p75NTR and Nestin positive and GFAP negative. These co-cultured ASCs also expressed various functional markers of mature OECs: BDNF, GDNF and the myelin proteolipid protein (PLP). Thus, these results demonstrate that using specific scaffolds, ASCs might differentiate into OEC-like cells *in vitro* (99). Altogether, it can be inferred that OECs play a key role in cell differentiation toward a neural type and are able to prompt MSC differentiation towards neural phenotype and even to mature OECs. As such, these intrinsic properties of OECs may be relevant for therapeutic approaches aiming at CNS tissue regeneration.

## 4 OECs for tissue regeneration and transplantation

In recent years, OECs have been investigated for their reparative ability following acute or chronic lesions that involve CNS. As already mentioned, it appears that OECs may play a crucial role in the treatment of SCI (100–103). Usually, SCI severely affects CNS microenvironment, leading to a series of deleterious processes such as inflammation and hypoxia, and progressive cell death (104). OECs exhibit several characteristics that enable them to have beneficial effects in neuro-repairing potential. They are able to reduce the inflammatory response following injury, thereby decreasing the size of the glial scar and promoting angiogenesis. In addition, they promote regrowth, plasticity and remyelination of axons (105–107). OECs can also interact with resident cell populations, particularly astrocytes and meningeal cells, either within the window of the glial scar formation or once the scar has already established (108, 109). Thus, in addition to penetrate glial tissue, OECs also produce extracellular matrix proteases and can reduce astrocytic reactivity. Overall, these properties may reduce glial scar formation and all consequential limitations, which strongly limit axonal regrowth and injury bridging (110). In SCI animal models, grafted OECs exhibit the ability to promote axon regrowth and propagation (30, 100, 111). In particular, OEC transplantation improves sensorimotor and autonomic nerve recovery, also reducing neuropathic pain due to SCI. OECs secrete a number of neurotrophic factors, which allow the establishment of a favorable microenvironment for the regrowth of damaged axons. Undoubtedly, it is crucial to have functional recovery in transplanted animals in order to consider OEC transplants a successful therapy for the treatment of SCI (107). A study of Ramon-Cueto et al., revealed that adult rats undergoing spinal cord resection and subsequent OEC transplantation, showed both functional and structural recovery. In particular, from 3 to 7 months after surgery, all transplanted animals improved locomotor functions and sensorimotor reflexes (103). To show actual recoveries in transplanted animals, electrophysiological studies were also carried out, demonstrating that animals with transplanted OECs not only exhibited functional recovery, but also showed recovery of action or evoked potentials (112, 113). Studies on OEC transplantation have also been carried out in human clinical in many countries around the world. Completed clinical trials have demonstrated the safety and efficacy of OEC transplantation, but recovery in patients is often highly variable. This variability may be related to a number of factors, such as difficulties in establishing master cell banks and working cell banks, cell purity, and transplantation techniques. Forty-four eligible trials, involving 1,266 SCI patients, investigated several cell-based treatments to improve functional independent misure (FIM) score. Among them, OEC transplantation proved to ameliorate the FIM score at 6 months, thus improving disease prognosis (114). However, a common consequence in these studies is the poor survival of transplanted cells, with survival rates ranging from 0.3 percent to 3 percent. This issue is probably related to the fact that when OECs are isolated, expanded *in vitro*, and then transplanted into the injury site, their therapeutic potential is reduced, probably due to bleeding, damaged tissue and anatomical structures and hostile microenvironment present in the lesioned area (115–117). Therefore, *in vitro* models challenging OECs, by mimicking the injured tissue microenvironment, are needed. For example, it has been found that preconditioned OECs showed increased migratory, phagocytic and immunomodulatory capacities. To improve their efficacy and yield upon graft, cells could be then exposed to a low oxygen level, or they could grow into three-dimensional scaffolds before being transplanted into the lesion (116).

Another effective strategy for SCI treatment is the co-transplantation of NSCs and OECs. Indeed, co-grafting of NSCs and OECs ameliorate SCI by inhibiting receptor-interacting protein kinase 3 (RIP3)/mixed lineage kinase domain-like protein (MLKL)-mediated necroptosis and stimulating NSC proliferation in the medulla. Evidence reports that OECs are able to increase NSCs proliferation and differentiation and, importantly, co-grafts significantly support NSCs survival, opening the way for a potential stem cells-based regenerative approach. In this way, neural regeneration could be improved exploiting the synergistic effect of NSCs and OECs (118). In addition, a study by He Y. t al., showed that curcumin-activated OECs (aOECs) effectively improve neuronal differentiation of NSCs even under conditions of inflammation, and co-transplantation of aOECs and NSCs enhaces the neurological recovery of rats after SCI, providing a hopeful strategy for SCI repair by co-transplantation of aOECs and NSCs (119). Besides OEC transplantation, OECs-CM has also been shown to have therapeutic effects for SCI, enhancing functional recovery and axonal regeneration probably because of various factors previously secreted by OECs in their culture medium (120). In this context, exosomes derived from OECs (OEC-Exo) also promote neuronal survival and improve axon condition, facilitating functional recovery following SCI. OEC-Exo can be internalized by microglia/macrophages and are able to modulate their polarization. The main ability of OEC-Exo consists in an immunomodulatory function that shape immune microenvironment towards a pro-regenerative phenotype, supporting OEC-Exo as neuroprotective and regenerative strategy for CNS diseases (121). Furthermore, OECs secrete, *via* exosomes, alpha B-crystallin (CryAB), an anti-inflammatory protein, leading to an intercellular immune response. Thus, CryAB, together with other OEC-secreted factors, may ameliorate the hostile growth environment created by neurotoxic reactive astrocytes following CNS injury (122).

SCI microenvironment is characterized by a prevalence of M1-like pro-inflammatory macrophages over M2-like. This phenomenon results in a microenvironment that is unfavorable for cell differentiation and regeneration. Therefore, for a better potential regenerative strategy, a fundamental role is played by immune cell modulation (123, 124). Macrophages are a prominent population in SCI microenvironment, also able to alter the activity of transplanted OECs. However, the interaction appears to be reciprocal, as OECs express MIFs and can also lead to reduced macrophage recruitment *in vitro* (45). To enhance this interaction in favor of OECs by improving the cellular microenvironment at the injury level, a study of vascular endothelial growth factor (VEGF) and platelet-derived growth factor (PDGF) modulation in the SCI microenvironment was carried out. It was shown that CM from macrophages exposed to PDGF or combined VEGF and PDGF, under inflammatory conditions, increased OEC phagocytosis, also modulating the expression of genes related to nerve repair. Specifically, both PDGF and VEGF/PDGF reduced pro-inflammatory cytokines (i.e., TNF) by decreasing NF-κB translocation, promoting phagocytosis of myelin debris. For this reason, administering growth factors before OEC transplantation could improve transplant success and neural recovery (125).

## 5 Conclusions

Our knowledge of the properties, functions, and therapeutic potential of OECs is markedly increasing. OECs can be considered as a good candidate for cell-replacement and have shown remarkable capabilities to exert neuroprotective mechanisms. The uniqueness of OECs appears to collaborate with other recruited cell types to orchestrate the molecular signaling responsible for resolving the inflammatory state and creating a favorable environment for neural regeneration.

However, the development of human OEC transplants for clinical application in SCI still requires an in-depth understanding of the cellular and molecular biological characteristics of OECs. It seems now clear that OECs expanded *in vitro* and grafted back *in vivo* show limited therapeutic potential, probably due to the hostile microenvironment at the damaged tissue. In fact, a major issue limiting spinal cord regeneration is also the poor survival of transplanted cells (126). In order to describe the therapeutic potential of OECs it appears critical to characterize OEC gene expression aiming at identifying OEC-specific markers. Indeed, the most used marker to identify OECs, p75NTR, is also expressed *in vitro* by Schwann cells (10), astrocytes and lamina propria MSCs (127). The lack of a solid method for OECs identification, isolation and purification is among the main factors limiting reproducibility and reliability of transplantation studies. Furthermore, without a unique method for OEC identification, it is possible that their repair capacity is influenced by the presence of the various cells types co-existing alongside OECs.

One of the most effective approaches in transplantation of OECs is co-grafting with NSCs, achieving better therapeutic effects. Indeed OECs, by releasing trophic factors into the microenvironment, also play an important role in promoting the differentiation of NSCs, able to change their morphology, stimulating their differentiation towards mature neurons.

Despite the variability of results reported and limiting factors, OECs should be considered as valuable cell-based approach for SCI and a potential candidate to promote cell differentiation and regeneration. Finally, a deeper understanding of OEC anti-inflammatory properties and their interplay with other cells involved in neuro-repairing is crucial for the development of future therapies, using transplantation of OECs to treat neural injuries.

## Author contributions

Conceptualization: SD, SD’A, NV, and RP; writing—original draft preparation: SD, SD’A, NV, and RP; writing—review and editing: all authors; visualization: SD, SD’A, CA, AP, and FT; supervision: DLF, AZ, RG, DT, GLV, NV, and RP. All authors contributed to the article and approved the submitted version.
